# Mercury in Precipitation at an Urbanized Coastal Zone of the Baltic Sea (Poland)

**DOI:** 10.1007/s13280-014-0494-y

**Published:** 2014-02-26

**Authors:** Dominika Saniewska, Magdalena Bełdowska, Jacek Bełdowski, Lucyna Falkowska

**Affiliations:** 1 Institute of Oceanography, University of Gdańsk, Av. Marszałka Piłsudskiego 46, 81-378 Gdynia, Poland; 2 Institute of Oceanology, Polish Academy of Sciences, Street Powstańców Warszawy 55, 81-712 Sopot, Poland

**Keywords:** Hg, Precipitation, Wet deposition, Sources

## Abstract

Wet deposition is an important source of metals to the sea. The temporal variability of Hg concentrations in precipitation, and the impact of air masses of different origins over the Polish coastal zone were assessed. Samples of precipitation were collected (August 2008–May 2009) at an urbanized coastal station in Poland. Hg analyses were conducted using CVAFS. These were the first measurements of Hg concentration in precipitation obtained in the Polish coastal zone. Since Poland was identified as the biggest emitter of Hg to the Baltic, these data are very important. In the heating and non-heating season, Hg concentrations in precipitation were similar. Hg wet deposition flux dominated in summer, when the production of biomass in the aquatic system was able to actively adsorb Hg. Input of metal to the sea was attributed to regional and distant sources. Maritime air masses, through transformation of Hg(0), were an essential vector of mercury in precipitation.

## Introduction

Mercury (Hg) is considered as a particularly dangerous pollutant among the many present in the Baltic Sea. It is highly neurotoxic, and produces also mutagenic, embryotoxic, nephrotoxic, and allergic effects in organisms. The aquatic environment is particularly susceptible to Hg pollution, where it can be accumulated and biomagnified in the food chain, representing a real risk for human health (Kabata-Pendias and Mukherjee 2007). An important source of Hg to the sea is wet deposition.

Wet deposition is very efficient in atmosphere cleanup. It is estimated to remove 70–80 % of aerosols mass from the atmosphere in temperate zones (Falkowska and Lewandowska 2009). According to GRAHM and GEOS-CHEM Models, a major part of Hg wet deposition originates from atmosphere cleanup above the marine and planetary boundary layer (Dastoor and Larocque 2004; Selin and Jacob 2008). As a consequence, the majority of Hg in wet deposition does not originate from local sources, but from the global mass undergoing long range transport. The contribution of wet to total atmospheric deposition of mercury depends mostly on amount, intensity, and frequency of precipitation, and on the concentration of various physical and chemical mercury species in the air (Lindberg and Stratton 1998).

Despite numerous studies in countries of Western and Southern Europe, and in Scandinavia, information about Hg levels in other parts of Europe is still missing. As a consequence, it is impossible to predict the environmental and health impact of mercury pollution on the European scale. Poland is leading the list of Hg-emitting countries (AMAP/UNEP 2008). It is also considered as a major Hg emitter to the Baltic Sea (Bartnicki et al. 2012); however, there are no studies to support this statement. Although estimated loads of Hg to the Baltic are available in the literature (Wrembel 1997; Szefer 2002), they are based on the studies conducted in the 80s and 90s of the twentieth century, and should be validated. According to the latest reports of EMEP and HELCOM (Bartnicki et al. 2010, 2011, 2012), atmospheric deposition of Hg to the Baltic Sea from Poland is several times higher than that of other Baltic Countries. Those estimations are, however, not based on the data recorded from sampling stations in Poland. That is why the aim of this study was the estimation of mercury concentration in wet deposition in the coastal zone of the southern Baltic, as well as the determination of factors influencing its magnitude. In addition, the contribution of local/regional/remote sources to mercury deposition in the southern Baltic region was assessed.

## Materials and Methods

### Hg in Precipitation

**Fig. 1:**
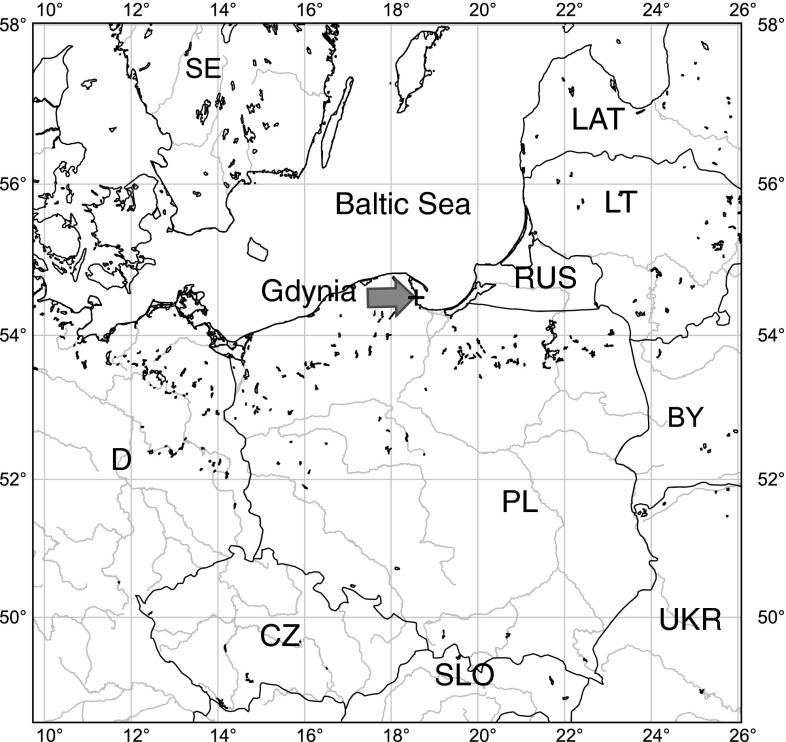
Sampling location

Samples of precipitation were collected at the coastal station situated on the roof of the building of the Institute of Oceanography University of Gdańsk in Gdynia (54°30′34″N, 18°32′28″E) (Fig. 1). The height of the building (20 m AGL) enabled sampling above the treetops and nearby buildings. The Institute is located 800 m from the sea, at the urbanized region (city center) in the immediate vicinity of the main routes.

Samples were collected from August 2008 to May 2009 with the bulk collector composed of a Teflon funnel of 20-cm diameter (reception area of 0.0314 m^2^) directly connected to a borosilicate glass bottle. To reduce the transformations taking place in collected sample and to avoid sample contamination, the collector was exposed before and changed immediately after precipitation. In each case, the amount of precipitation was measured (mm). Until analysis, samples conserved with nitric acid were stored at 4 °C. Once a week, a blank sample was collected. For this purpose, deionized water with a controlled Hg concentration was poured through the collector. Concentrations reported in this study have been blank-corrected by subtracting the blank value. The method detection limit (3 SD blank samples) equaled 0.05 ng dm^−3^.

Analysis of total mercury concentration in precipitation was conducted by means of CV-AFS (TEKRAN 2600). According to US EPA method 1631 (US EPA 2002), samples were oxidized by the addition of BrCl and pre-reduced with hydroxylamine hydrochloride solution 1 h prior to the analysis. Quality control procedures for water samples included blanks and deionized water spiked with mercury nitrate in the range from 0.5 to 25 ng dm^−3^, and produced adequate precision (1 % RSD) and recovery (98–99 %). Quality control procedures (three replicate samples, analysis of reference materials BCR-579—coastal sea water) revealed that the measurement uncertainty was below 5 %. The limit of quantification for the water samples was 0.05 ng dm^−3^.

Hg concentration in precipitation was used for the calculation of wet deposition fluxes:

1 $$ {F_{\text{wet}}} = CR, $$

where *F*_wet_ is the wet deposition fluxes (ng m^−2^); *C* the Hg concentration in precipitation (ng dm^−3^); and *R* is the precipitation amount (mm = dm^3^ m^−2^).

Precipitation was collected for 10 months (August 2008–May 2009); in order to calculate the annual wet deposition flux, values of wet deposition fluxes were estimated for 2 months (June and July 2008). The calculations were based on the monthly precipitation amount measured at the station of the Institute of Meteorology and Water Management in Gdynia (Miętus et al. 2012a, b) and an average Hg concentration in the precipitation in August 2008 and May 2009.

During the experiments continuous recording of meteorological parameters (air temperature—*T*_a_, atmospheric pressure—*P*_a_, wind speed—*V*_w_, wind direction—*W*_d_, relative humidity—RH and precipitation amount—*R*) was performed by means of HUGER WEATHER STATION.

The Foundation: Agency of Regional Air Quality Monitoring in the Gdańsk metropolitan area provided the results of ozone and solar radiation intensity (www.armaag.gda.pl).

Based on the meteorological data and information when the heat and power plants operation starts and ends, two sampling periods were distinguished—heating season (from October to April) and non-heating season (from May to September).

Backward trajectories of air masses were determined by means of the HYSPLIT (Hybrid Single Particle Lagrangian Integrated Trajectory) model. HYSPLIT is a complete system for computing simple air parcel trajectories to complex dispersion and deposition simulations. The detailed model description can be found at the model webpage: http://ready.arl.noaa.gov/HYSPLIT.php (Draxler and Rolph 2003; Rolph 2003). Details were presented in Bełdowska et al. (2012). On the basis of backward trajectories, air masses were divided into two sectors: maritime (NNW-N-NNE) and continental (NWW-W-SWW-SSW-S-SSE-SEE-E-NEE).

### Hg in Particulate Matter

The interpretation of the results takes into account the concentration of Hg in particulate matter in air in Gdynia, from the same studied period, as described in Bełdowska et al. (2012).

## Results

**Fig. 2:**
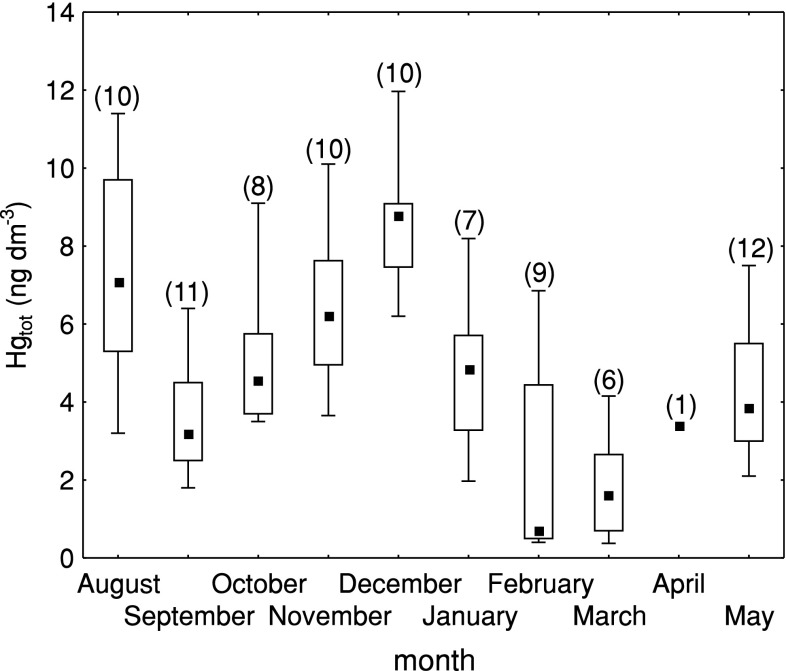
Statistical characteristics of total mercury (Hg_tot_) concentration in precipitation at Gdynia station (August 2008–May 2009), *in brackets* the number of samples

From August 2008 to the end of May 2009, 84 samples of rain were collected, at the coastal station in Gdynia. Hg concentration varied from 0.4 ng dm^−3^ (02.03.2009) to 12.0 ng dm^−3^ (24.12.2008) (Fig. 2). The mean concentration of Hg amounted to 5.0 ng dm^−3^, and median value to 4.7 ng dm^−3^. Taking into account individual months, the highest Hg concentrations were measured in the precipitation during the warmest days of the vegetation period—in August (median 7.1 ng dm^−3^) and in the middle of the heating season in December (median 8.8 ng dm^−3^), whereas the lowest concentration of mercury occurred in February and March 2009 (median 0.7 and 1.6 ng dm^−3^, respectively). In the whole experiment period, Hg concentration in the range up to 6 ng dm^−3^ predominated, which represents around 64 % of the collected data. Hg concentration over 10 ng dm^−3^ in precipitation contributed to less than 5 % of the results.

**Fig. 3:**
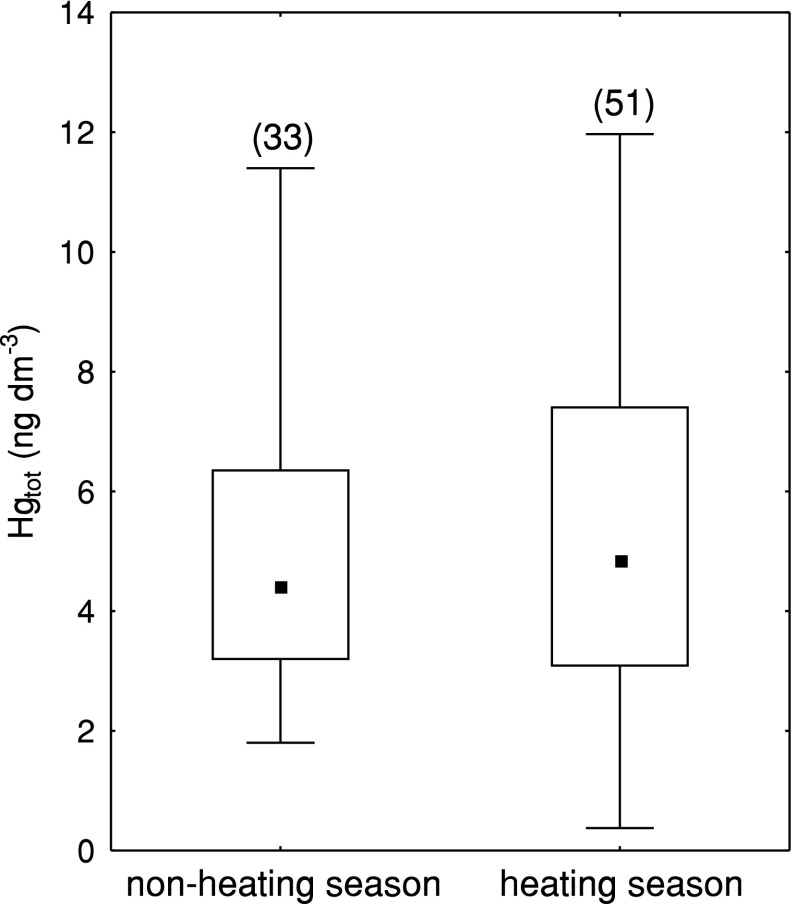
Statistical characteristics of total mercury (Hg_tot_) concentration in precipitation in the non-heating season (May–September) and in the heating season (October–April), at Gdynia station, *in brackets* the number of samples

During the heating season, more days with rain occurred (51 of 84). Despite this difference in the occurrence of precipitation, Hg concentrations in the non-heating and heating seasons were similar (respective medians non-heating season 4.4 ng dm^−3^; heating season 4.8 ng dm^−3^) (Fig. 3). The heating season was characterized with a slightly wider range of concentrations of Hg in the precipitation (non-heating season 9.6 ng dm^−3^; heating season 11.6 ng dm^−3^).

In collected samples, the precipitation amount varied between 0.1 and 21.7 mm, during the study period, at the Gdynia station. An average precipitation amount was 5.0 mm and median 3.5 mm. The lowest values were observed in the period from November 2008 to January 2009, and the highest in August and at the beginning September. In contrast to concentrations of Hg in rain, precipitation amounts differ significantly in both seasons. The sum of precipitation in the non-heating season was somewhat higher (326.2 mm season^−1^) than in the heating season (193.9 mm season^−1^).

The wet deposition flux varied from 0.3 ng m^−2^ (February) to 165.1 ng m^−2^ (August). The average during the measuring campaign was 26.3 ng m^−2^ and median 11.8 ng m^−2^. In the non-heating season (1784.5 ng m^−2^ season^−1^), deposition of Hg to the coastal zone of the southern Baltic was higher than in the heating season (989.3 ng m^−2^ season^−1^).

## Discussion

**Fig. 4:**
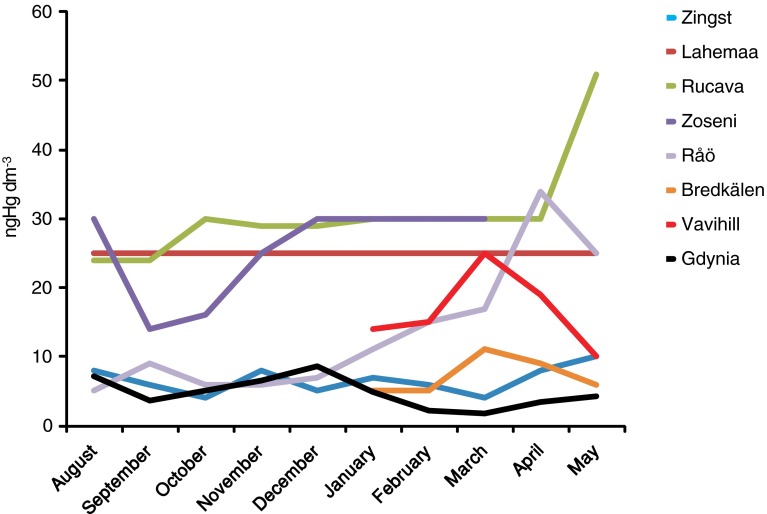
Mercury concentration in precipitation (August 2008–May 2009) at Gdynia station and at HELCOM sites (according to Appendix A: Bartnicki et al. 2010, 2012)

Hg concentrations in precipitation measured in the years 2008–2009 at the sampling stations in Gdynia (Fig. 2) were substantially lower than those measured in other urbanized and industrialized regions of Poland (Pszczyna: 16–110 ng dm^−3^), at the Lithuanian coast (Preila: 10 ng dm^−3^; 3–30 ng dm^−3^) or in the Great Lakes region (10–60 ng dm^−3^) (Milukaite et al. 2008; Ebinghaus et al. 2009; Zielonka and Nowak 2010). They were similar to values observed in similar periods at other stations on the Baltic coast: in Germany (Zingst: range 2.5–20.6 ng dm^−3^; median 6.5 ng dm^−3^), Sweden (Råö: range 4.9–41.0 ng dm^−3^; median 9.5 ng dm^−3^), and Finland (Virolahti II: range 2–19 ng dm^−3^; median 4 ng dm^−3^) (www.ebas.nilu.no). Taking into account HELCOM reports (Bartnicki et al. 2010, 2011) that present data concerning monthly mean concentration of Hg in precipitation in 2008 and 2009 at in the Baltic coastal stations, it is evident that concentrations and variability observed at Gdynia station are most similar to those recorded at Zingst station, and several fold lower than those recorded at Lithuanian and Estonian stations (Appendix A in: Bartnicki et al. 2010, 2011) (Fig. 4). Especially in August, October, and November, Hg concentrations in precipitation in Gdynia and Zingst were similar—when air masses incoming to Gdynia originated in the Zingst region (SWW, W). These masses of air have not been contaminated by Hg at SWW, W region of Poland. Based on data available in Appendix A in HELCOM reports from the years 2008 and 2009 (Bartnicki et al. 2010, 2011), the magnitude of wet deposition of Hg was estimated at Zingst station, for period August–December 2008: 1706 ng m^−2^ while for the period January–May 2009: 1157 ng m^−2^. These values were similar to those observed in Gdynia: 1742 ng m^−2^ for the period August–December 2008 and 472 ng m^−2^ for the period January–May 2009. The values of mercury deposition in the Polish coastal zone reported by HELCOM for 2008 and 2009 were, however, several times higher than those for the Zingst area. This suggests that data concerning deposition of Hg for the Polish coastal zone are overestimated using HELCOM reports, in which Hg flux for the Tri-City Agglomeration varied between 7.3 and 11.0 g Hg km^−2^ year^−1^ (Bartnicki et al. 2010). Observed differences might be a result of the improper reporting, rather than changes in the environment. Performance of the model used by EMEP/HELCOM for wet deposition data is tested with several monitoring stations, but none of those stations is located in Poland. Data for the Polish coast are solely model-based—and this first comparison with environmental data shows that the model needs calibration in this area, and deposition values will probably need recalculation.

### Seasonal Variability of Hg Concentration in Precipitation

Hg concentration in precipitation did not show seasonal variability (Mann–Whitney *U* test *p* = 0.80), and the maximum values observed in precipitation in both heating and non-heating seasons were similar (Fig. 3). This means that in both seasons, an Hg source existed in the coastal zone of the Gulf of Gdańsk, which controlled the concentrations in the precipitation.

An essential role was also played by the chemical composition of maritime and terrestrial air masses moving over Gdynia. During the heating season, no significant differences in Hg concentration in precipitation (Hg(wet)) were observed between maritime and terrestrial air masses (Mann–Whitney *U* test, *p* = 0.74), but the Hg origin was different. High Hg concentration in continental air masses was caused mostly by elution of particulate Hg(p) originating from coal combustion (Hg(wet)/Hg(p) *r* = 0.61, *p* = 0.01) (Hławiczka et al. 2006; Wängberg et al. 2008). Precipitation was more effective in eluting Hg in coarse particles (*r* = 0.66, *p* = 0.01) than in fine particles (*r* = 0.51, *p* = 0.03). In maritime air masses, characterized by smaller than continental Hg(p) concentration, no correlation between Hg concentration in precipitation and in particles was observed (*r* = 0.17, *p* = 0.71). This suggests that Hg(p) was not the source of mercury wet deposition. In maritime air masses, Hg concentration depended on temperature (*r* = 0.74, *p* < 0.01) and relative humidity of air (*r* = −0.63, *p* = 0.03), which could indicate transformation of Hg(0) to reactive gaseous mercury (RGM) (Malcolm et al. 2003; Poissant et al. 2005). The lack of correlation of Hg(wet) with Hg(p) and the inverse proportional relation to air humidity suggest, that RGM to a small extent was adsorbed on particles, most of it was readily eluted by means of precipitation. During low wind speeds (*V*_w_ < 4 m s^−1^), and for air masses moving at altitudes below 500 m, Hg concentration in precipitation increased proportionally to ozone concentration (*r* = 0.77, *p* = 0.04) and solar radiation (*r* = 0.80, *p* = 0.03). This suggests that in the ozone-polluted coastal zone of the Gulf of Gdańsk, solar radiation induced mercury transformation from gaseous to reactive form occurred. During strong winds (*V*_w_ > 4 m s^−1^), the highest values of mercury concentrations in precipitation were observed in air masses moving at altitude >1500 m from the Norwegian Sea Area. Northern Scandinavia is considered a clean area, with the lowest atmospheric mercury concentrations in Europe (Ebinghaus et al. 2009). This suggests that mercury measured in the air masses from Northern Europe originated from a global long range mercury transport. Holmes et al. (2009) reported that the air from the upper troposphere can be a significant source of RGM in the marine boundary layer, and the reactive mercury contribution can amount to 40 %.

Independently from the air mass origin, two times lower median concentrations of mercury were measured in snow as compared to rain (Mann–Whitney *U* test, *p* < 0.01). This is in agreement with previous studies, which showed that snow is less efficient in atmospheric mercury elution (Landis et al. 2002; Ebinghaus et al. 2009). Significant influence on the rate of atmospheric Hg removal was observed for low air temperature, which slows down Hg transformations in atmosphere (Landis et al. 2002). The lowest concentrations in precipitation were recorded in February and March 2009, when icing of the Baltic restricted aerosol formation. In this period, the existence of snow cover additionally restricted mercury input from terrigenous sources.

During the warm season Hg concentrations in precipitation originating from both continental and maritime air, masses did not differ significantly (Mann–Whitney *U* test, *p* = 0.20). In both cases mercury concentration in precipitation depended on air temperature; however, in maritime air, this dependency was stronger (*r* = 0.86, *p* = 0.01) than in continental air (*r* = 0.59, *p* < 0.01). This indicates that in warm maritime air Hg transformations to reactive form were faster than in continental air. In maritime air masses precipitation eluted mostly Hg in coarse marigenic particles (*r* = 0.64, *p* = 0.04), which were formed in air masses traveling low (<500 m) over the open sea (Hg_tot_/*V*_w_: *r* = 0.69, *p* = 0.04). In the air coming from land, precipitation eluted mostly mercury associated with fine particles (*r* = 0.57, *p* = 0.03), which originated from anthropogenic sources or after evaporation of Hg from coarse particles.

### Wet Deposition of Hg

**Fig. 5:**
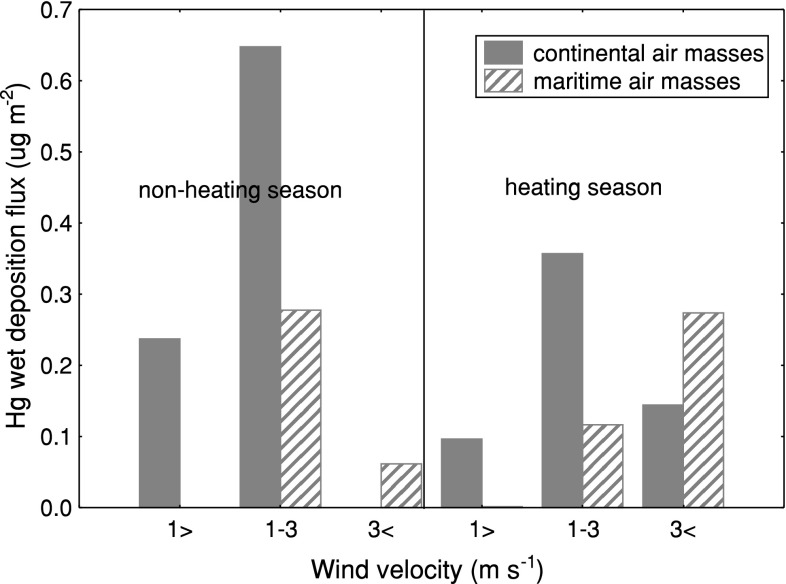
Influence of wind speed and air mass on the Hg wet deposition flux in Gdynia

The area of the Gulf of Gdańsk is dominated by westerly winds (S, SW, W, NW). Their total annual contribution varies from 40 to 60 %. Easterly and southerly winds are less frequent (30–40 %). Winds from N, NE, and E sectors are rare (<10 %) (Majewski 1990; Miętus and Sztobryn 2011). The largest precipitations occur in July or August, and the smallest in March or April (Majewski 1990; Miętus and Sztobryn 2011). Taking into account the above-mentioned multiannual observations, wet deposition in particular seasons was estimated, including the origin of air masses. A key role in Hg wet deposition during the warm season was played by the regional sources (1 m s^−1^ ≤ *V*_w_ < 3 m s^−1^), both terrestrial and marine, which in total contributed to 75 % of Hg deposited within the season (0.9 μg m^−2^) (Fig. 5). The regional sources (1 m s^−1^ ≤ *V*_w_ < 3 m s^−1^), were also important during the warm season, contributing 20 % of mercury deposited in that season (Fig. 5). In the case of both local and regional sources, precipitation was formed in low altitude air masses (<500 m). This suggested that the high wet deposition flux of Hg in summer months resulted from oxidation of elemental mercury to its reactive form and its subsequent elution with precipitation. This process is especially intense in the polluted coastal zone, where apart from halogens also tropospheric ozone could induce Hg oxidation. Only 5 % of the mercury deposited in Gdynia came from distant sources (*V*_w_ > 3 m s^−1^) (Fig. 5). In this case, mercury was removed from air masses at high altitudes (>1000 m) traveling from northern Europe. During the heating season local sources played a marginal role in mercury wet deposition in the coastal zone of the Gulf of Gdańsk (10 % of deposited Hg). A key role was played by the regional and distant sources, which were responsible for 48 and 42 % of the mercury deposited in Gdynia, respectively (Fig. 5). Hg from the land sector predominated in the regional Hg deposition. In this case, mercury originated mostly from fossil fuel combustion in power plants. In remote sources Hg from the marine sector dominated. This is a consequence of the fact that during the heating season, emission from regional anthropogenic sources matched that of remote sources.

## Conclusions

Hg in precipitation in the Polish coastal zone of the Baltic was not characterized by seasonal variability. Concentrations of Hg in precipitation in both seasons were similar. High concentrations of Hg observed during winter were caused by the intense combustion of coal, both in heat and power plants as well as in household heating. A peak observed during summer was caused by the transformation of insoluble elemental Hg into its reactive form in the coastal zone. Such a situation occurs when polluted terrestrial air masses meet humid, halogen-rich marine air masses, which is a common situation in this area (Lewandowska and Falkowska 2013a, b), Hg(0) was most probably oxidized by the halogen radicals to Hg(II) and adsorbed on the condensation nuclei, being subsequently washed out from the atmosphere during rainfall. In consequence, the highest monthly deposition of Hg was observed in summer, when the production of biomass in the aquatic system was able to accumulate the Hg, and hence introduce it into biogeochemical cycle in the coastal zone of the southern Baltic Sea. Maritime air masses, through transformation of Hg(0), were essential vector of mercury in precipitation.

In the heating and non-heating seasons, an important role was played by the regional sources in wet deposition of Hg. During the heating season, terrestrial regional and distant sources were dominant and accounted for 48 and 42 % of the mercury deposited in Gdynia. In warm months, both terrestrial and marine regional sources played a major role and were responsible for more than 75 % of Hg deposited in that season.

These measurements indicate that because of the lack of sampling stations in the Polish coastal zone, values from HELCOM reports are significantly overestimated. Verification of the atmospheric model with measurements conducted in Zingst in Germany and Råö in Sweden, although the distance is not large, seem insufficient to properly reproduce mercury wet deposition fluxes in the study area, which makes model calculations for the Polish coast questionable.
